# RIDDLE: Race and ethnicity Imputation from Disease history with Deep LEarning

**DOI:** 10.1371/journal.pcbi.1006106

**Published:** 2018-04-26

**Authors:** Ji-Sung Kim, Xin Gao, Andrey Rzhetsky

**Affiliations:** 1 Department of Computer Science, Princeton University, Princeton, New Jersey, United States of America; 2 King Abdullah University of Science and Technology (KAUST), Computational Bioscience Research Center (CBRC), Computer, Electrical and Mathematical Sciences and Engineering (CEMSE) Division, Thuwal, Saudi Arabia; 3 Institute for Genomics and Systems Biology, Computation Institute, Departments of Medicine and Human Genetics, University of Chicago, Chicago, Illinois, United States of America; Mines ParisTech, FRANCE

## Abstract

Anonymized electronic medical records are an increasingly popular source of research data. However, these datasets often lack race and ethnicity information. This creates problems for researchers modeling human disease, as race and ethnicity are powerful confounders for many health exposures and treatment outcomes; race and ethnicity are closely linked to population-specific genetic variation. We showed that deep neural networks generate more accurate estimates for missing racial and ethnic information than competing methods (e.g., logistic regression, random forest, support vector machines, and gradient-boosted decision trees). RIDDLE yielded significantly better classification performance across all metrics that were considered: accuracy, cross-entropy loss (error), precision, recall, and area under the curve for receiver operating characteristic plots (all *p* < 10^−9^). We made specific efforts to interpret the trained neural network models to identify, quantify, and visualize medical features which are predictive of race and ethnicity. We used these characterizations of informative features to perform a systematic comparison of differential disease patterns by race and ethnicity. The fact that clinical histories are informative for imputing race and ethnicity could reflect (1) a skewed distribution of blue- and white-collar professions across racial and ethnic groups, (2) uneven accessibility and subjective importance of prophylactic health, (3) possible variation in lifestyle, such as dietary habits, and (4) differences in background genetic variation which predispose to diseases.

## Author summary

Race and ethnicity are typically unspecified in very large electronic medical claims datasets. Computationally estimating a patient’s missing race and ethnicity from their medical records is important on both an academic and practical basis. Academically, discriminative medical events tell us about racial and ethnic health disparities and divergent genetic predispositions. Practically, imputed race and ethnicity information can substantially improve genetic and epidemiological analyses with these large datasets.

## Introduction

Electronic medical records (EMRs) are an increasingly popular source of biomedical research data [1]. EMRs are digital records of patient medical histories, describing the occurrence of specific diseases and medical events such as the observation of heart disease or dietary counseling. EMRs can also contain demographic information such as gender or age.

However, these datasets are often anonymized and lack race and ethnicity information (e.g., insurance claims datasets). Race and ethnicity information may also be missing for specific individuals within datasets. This is problematic in research settings as race and ethnicity can be powerful confounders for a variety of effects. Race and ethnicity are strong correlates of socioeconomic status, a predictor of access to and quality of education and healthcare. These factors are differentially associated with disease incidence and trajectories. As a result of this correlation, race and ethnicity may be associated with variation in medical histories. As an example, it has been reported that referrals for cardiac catheterization are rarer among African American patients than in White patients [2]. Furthermore, researchers have reported differences in genetic variation which influence disease across racial and ethnic groups [3]. Due to the association between race, ethnicity and medical histories, we hypothesize that clinical features in EMRs can be used to impute missing race and ethnicity information.

In addition, race and ethnicity information can be useful for producing and investigating hypotheses in epidemiology. For example, variation in disease risk across racial and ethnic groups that cannot be fully explained by allele frequency information may provide insights into the possible environmental modifiers of genes [3].

### Imputation

The task of race and ethnicity imputation can be serialized as a supervised learning problem. Typically, the goal of imputation is to estimate a posterior probability distribution over plausible values for a missing variable. This distribution of plausible values can be used to generate a single imputed dataset (e.g., by choosing plausible values with highest probability), or to generate multiple imputed datasets as in *multiple imputation* [4]. In our setting, the goal was to impute the distribution of mutually-exclusive race and ethnicity classes given a set of clinical features. Features comprised age, gender, and codes from the International Disease Classification, version 9 (ICD9, [5]); ICD9 codes describe medical conditions, medical procedures, family information, and some treatment outcomes.

Bayesian approaches to race and ethnicity imputation using census data have been proposed [6] and have been used for race and ethnicity imputation in EMR datasets [7]. However, these approaches require sensitive geolocation and surname data from patients. Geolocation and surname data can be missing in anonymized EMR datasets (as in the datasets used here), limiting the utility of approaches which use this information.

### Deep learning

Traditionally, logistic regression classifiers have been used to impute categorical variables such as race and ethnicity [8]. However, there has been recent interest in the use of deep learning for solving similar supervised learning tasks. Deep learning is particularly exciting as it offers the ability to automatically learn complex representations of high-dimensional data. These representations can be used to solve learning tasks such as regression or classification [9].

Deep learning involves the approximation of some utility function (e.g., classification of an image) as a neural network. A neural network is a directed graph of functions which are referred to as units, neurons or nodes. This network is organized into several layers; each layer corresponds to a different representation of the input data. As the input data is transformed and propagated through this network, the data at each layer corresponds to a new representation of the sample [9]. For our imputation task, the aim was to learn the representation of an individual as a mixture of race and ethnicity classes where each class is assigned a probability. This representation is encoded in the final output layer of the neural network. The output of a neural network functions as a prediction of the distribution of race and ethnicity classes given a set of input features.

**Fig 1:**
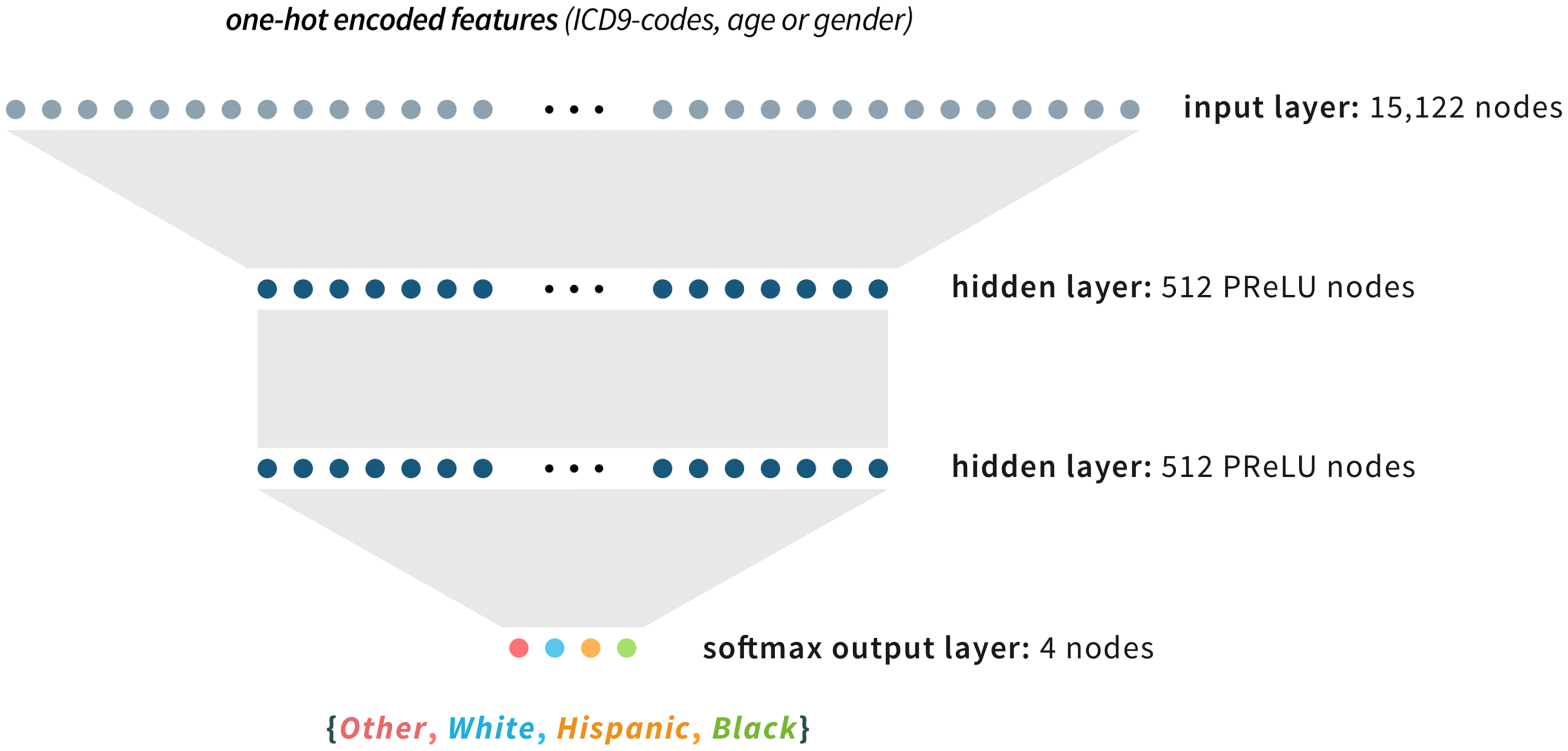
Neural network architecture. RIDDLE uses a multi-layer perceptron (MLP) network containing two hidden layers of either Rectified Linear Units (ReLU) or Parametric Rectified Linear Unit (PReLU) nodes. The input to the MLP is the set of binary encoded features comprising age, gender, and International Disease Classification, version 9 (ICD9) codes. The output is the set of probability estimates for each of the four race and ethnicity classes.

We introduce a framework for using deep learning to estimate missing race and ethnicity information in EMR datasets: **RIDDLE** or **R**ace and ethnicity **I**mputation from **D**isease history with **D**eep **LE**arning. RIDDLE uses a relatively simple multilayer perceptron (MLP), a type of neural network architecture that is a directed acyclic graph (see Fig 1).

In addition to investigating the novel utility of deep learning for race and ethnicity imputation, we used recent methods in interpreting neural network models [10] to perform a systematic evaluation of racial and ethnic patterns for approximately 15,000 different medical events. We believe that this type of large-scale evaluation of disease patterns and maladies by race and ethnicity has not been done heretofore.

## Results

We aimed to assess RIDDLE’s imputation performance in a multiclass classification setting. We used EMR datasets from Chicago and New York City, collectively describing over 1.5 million unique patients. There were approximately 15,000 unique input features consisting of basic demographic information (gender, age) and observations of clinical events (codified as ICD9 codes). The target class was race and ethnicity; possible values were White, Black, Other or Hispanic (see Table 1). Although race and ethnicity can be described as a mixture, our training datasets labeled race and ethnicity as one of four mutually exclusive classes. For the testing set, we treated the target race and ethnicity class as unknown, and compared the predicted class against the true class. The large dimensionality of features, high number of samples, and heterogeneity of the source populations present a unique and challenging classification problem.

**Table 1:** Race and ethnicity composition of the EMR dataset. The dataset comprised individuals from four race and ethnicity classes: Other, White, Hispanic, Black.

Ethnicity	Number of samples	Percent in dataset
Other	878,017	53.2%
White	308,323	18.7%
Hispanic	256,015	15.5%
Black	207,645	12.6%

In our experiments, RIDDLE yielded an average accuracy of 0.668, and cross-entropy loss of 0.857 on test data, significantly outperforming logistic regression, random forest classifiers, and gradient-boosted decision tree (GBDT) classifiers across all classification metrics (*p* < 10^−9^; see Table 2).

**Table 2:** Evaluation of RIDDLE and baseline classification methods. All values are averaged over ten *k*-fold cross-validation experiments. In addition, the precision, recall and ROC scores are averaged across classes, weighted by the number of samples in each class. Support vector machines (SVMs) could not be evaluated on the full dataset as individual trials required more than 36 hours of computation. For runtime comparisons a standard computing configuration was used: 16 Intel Sandybridge cores at 2.6 GHz and 16GB RAM; graphics processing units were not utilized.

Method	Accuracy	Loss	Precision	Recall	F1	Macro-average ROC	Runtime (h)
RIDDLE	**0.668**	**0.857**	**0.663**	**0.668**	**0.652**	**0.833**	0.962
logistic regression	0.644	0.928	0.639	0.644	0.611	0.807	**0.024**
random forest	0.629	0.962	0.641	0.629	0.578	0.799	2.395
GBDT	0.634	0.948	0.635	0.634	0.592	0.793	0.265
SVM, linear kernel	N/A	N/A	N/A	N/A	N/A	N/A	>36
SVM, polynomial kernel	N/A	N/A	N/A	N/A	N/A	N/A	>36
SVM, RBF kernel	N/A	N/A	N/A	N/A	N/A	N/A	>36

Support vector machines (SVMs) with various kernels were also evaluated. However, SVMs could not be feasibly used with the full dataset as individual trials took longer than 36 hours each (36 hours runtime was the allowed maximum on the system used in our analysis). Additional experiments involving a smaller subset of the full dataset (165K samples) were performed; in such experiments, SVMs could be practically utilized and RIDDLE significantly outperformed the baseline methods across all classification metrics (*p* < 10^−2^; see Table E in S1 Supplement).

**Fig 2:**
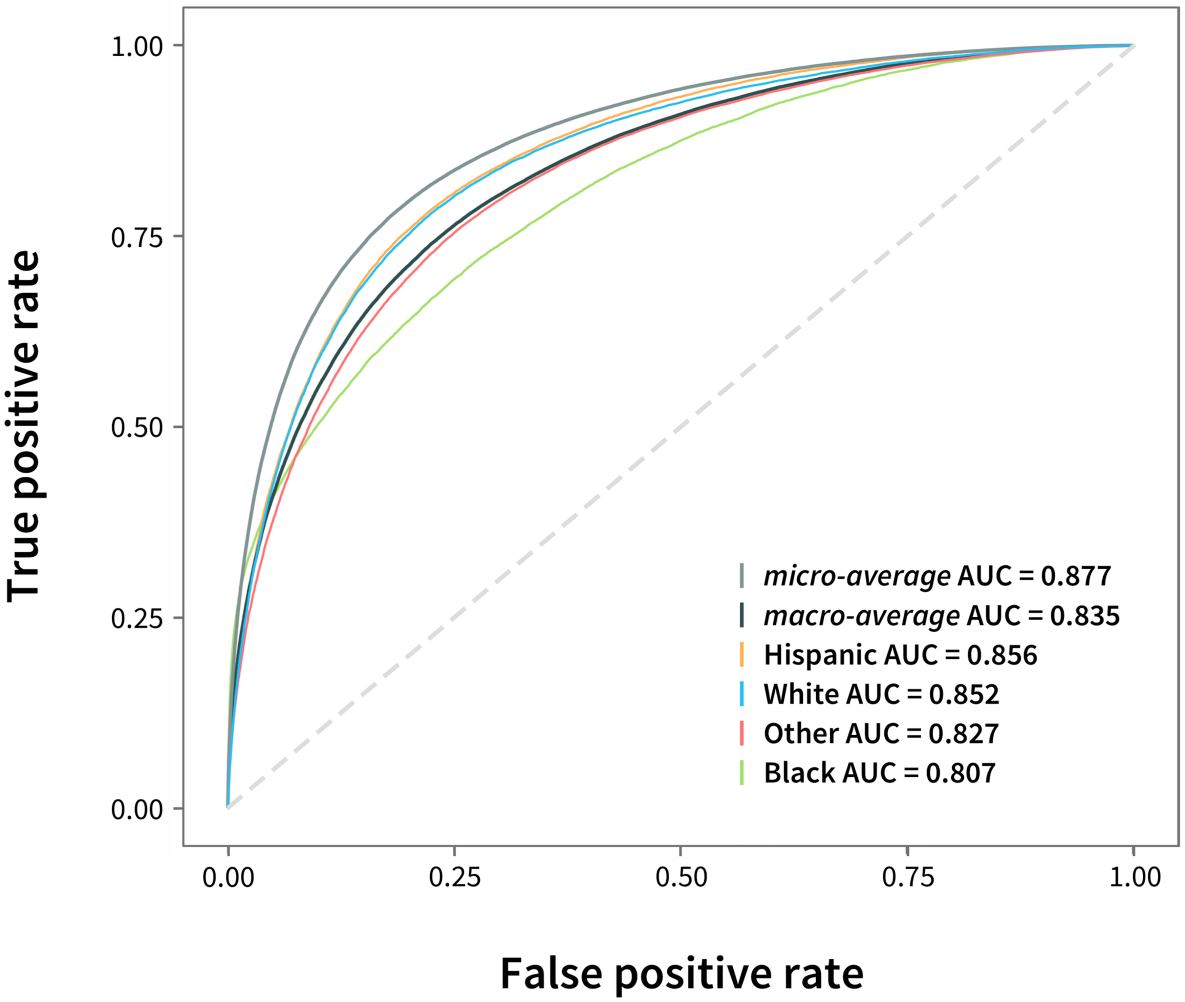
Receiver operating characteristic (ROC) curves. ROC curves and their corresponding area under the curve (AUC) values were calculated for each of the four race and ethnicity classes. Micro-average (all cases considered as binary, e.g., Hispanic vs. non-Hispanic) and macro-average (average across classes) curves were also computed. Data and metrics for a representative experiment is shown. Across experiments, the *mean* micro-average AUC was 0.874, and the macro-average AUC was 0.833.

While the multiclass learning problem appeared relatively hard, RIDDLE achieved class-specific receiver operating characteristic’s (ROC) area under the curve (AUC) values above 0.8 (see Fig 2), and a micro-average (all cases considered as binary) AUC of 0.874—significantly higher than that of logistic regression (mean = 0.854, *p* = 6.67 × 10^−11^), random forest (mean = 0.844, *p* = 2.05 × 10^−10^) and GBDT (mean = 0.846, *p* = 1.20 × 10^−10^) classifiers (see Table 2).

RIDDLE exhibited runtime performance comparable to that of other machine learning methods on a standard computing configuration without the use of a graphics processing unit or GPU (see Table 2).

As explained prior, SVMs were also evaluated but precise runtime measurements could not be obtained as the computational cost was too high. However, on a smaller subset (165K samples) of the full dataset where SVMs could be utilized, RIDDLE exhibited significantly faster runtime performance compared to all SVM methods (*p* < 10^−10^; see Table E in S1 Supplement).

### Influence of missing data on classifier performance

In order to replicate real-world applications where data other than race and ethnicity (e.g., features for specific samples) may be missing, we conducted additional experiments to simulate random missing data. A random subset of feature observations (ranging from 10% to 30% of all feature observations) was artificially masked completely at random.

Feature observations at the sample level (e.g., a particular ICD9 code for a specific patient) were randomly deleted to simulate random missing data. The number of whole features was kept fixed—only individual observations were removed. Otherwise, the same classification training and evaluation scheme was used as before. Under simulation of random missing data, RIDDLE significantly outperformed logistic regression, random forest classifiers and GBDTs in classification metrics across all simulation experiments (*p* < 10^−9^ for 10% and 20% missing data simulation, *p* < 10^−4^ for 30% missing data simulation; see Table 3).

**Table 3:** Evaluation of RIDDLE and other methods under simulation of random missing data. All values are averaged over ten *k*-fold cross-validation experiments involving different proportions of random missing data (10%–30%). In addition, the precision, recall and ROC scores are averaged across classes, weighted by the number of samples in each class. SVMs could not be evaluated on the full dataset as individual trials required more than 36 hours of computation.

Method	Accuracy	Loss	Precision	Recall	F1	Macro-average ROC
RIDDLE	**0.660**	**0.878**	**0.656**	**0.660**	**0.643**	**0.822**
logistic regression	0.639	0.941	0.634	0.639	0.604	0.800
random forest	0.623	0.978	0.635	0.623	0.567	0.789
GBDT	0.627	0.967	0.628	0.627	0.580	0.782
SVM, linear kernel	N/A	N/A	N/A	N/A	N/A	N/A
SVM, polynomial kernel	N/A	N/A	N/A	N/A	N/A	N/A
SVM, RBF kernel	N/A	N/A	N/A	N/A	N/A	N/A
(a) 10% missing data						
Method	Accuracy	Loss	Precision	Recall	F1	Macro-average ROC
RIDDLE	**0.654**	**0.897**	**0.649**	**0.654**	**0.631**	**0.814**
logistic regression	0.634	0.954	0.629	0.634	0.596	0.792
random forest	0.616	0.994	0.631	0.616	0.556	0.779
GBDT	0.622	0.979	0.624	0.622	0.572	0.774
SVM, linear kernel	N/A	N/A	N/A	N/A	N/A	N/A
SVM, polynomial kernel	N/A	N/A	N/A	N/A	N/A	N/A
SVM, RBF kernel	N/A	N/A	N/A	N/A	N/A	N/A
(b) 20% missing data						
Method	Accuracy	Loss	Precision	Recall	F1	Macro-average ROC
RIDDLE	**0.643**	**0.926**	**0.640**	**0.643**	**0.614**	**0.800**
logistic regression	0.629	0.968	0.623	0.629	0.587	0.784
random forest	0.610	1.009	0.625	0.610	0.545	0.769
GBDT	0.616	0.995	0.617	0.616	0.561	0.764
SVM, linear kernel	N/A	N/A	N/A	N/A	N/A	N/A
SVM, polynomial kernel	N/A	N/A	N/A	N/A	N/A	N/A
SVM, RBF kernel	N/A	N/A	N/A	N/A	N/A	N/A
(c) 30% missing data						

### Feature interpretation

A major criticism of deep learning is the opaqueness of trained neural network models for intuitive interpretation. While intricate functional architectures enable neural networks to learn complex tasks, they also create a barrier to understanding how learning decisions (e.g., classifications) are made. In addition to creating a precise race and ethnicity estimation framework, we sought to identify and describe the factors which contribute to these estimations. We computed DeepLIFT (Deep Learning Important FeaTures) scores to quantitatively describe how specific features contribute to the probability estimates of each class. The DeepLIFT algorithm compares the activation of each node to a reference activation; the difference between the reference and observed activation is used to compute the contribution score of a neuron to a class (see the Methods) [10].

If a feature contributes to selecting *for* a particular class, this feature-class pair is assigned a positive DeepLIFT score; conversely, if a feature contributes to selecting *against* a particular class, the pair is assigned a negative score. The magnitude of a DeepLIFT score represents the strength of the contribution.

Using DeepLIFT scores, we were able to construct natural orderings of race and ethnicity classes for each feature, sorting classes by positive to negative scores. The following example ordering shows how the example feature (heart disease) is a strong predictor for the African American class, and a weak (or negative) predictor for the Other class.

$$\begin{array}{c} \begin{array}{cc} \text{heart}\;\text{disease}\to \text{Other,}\; & \text{score}=-500\\ \text{heart}\;\text{disease}\to \text{White,}\; & \text{score}=-100\\ \text{heart}\;\text{disease}\to \text{Hispanic,}\; & \text{score}=+200\\ \text{heart}\;\text{disease}\to \text{Black,}\; & \text{score}=+500 \end{array}\;\Rightarrow \;\text{Black}>\text{Hispanic}>\text{White}>\text{Other} \end{array}$$

We computed the class orderings for all ∼15,000 features (see S1 Data). The orderings of the 10 most predictive features (by highest ranges of DeepLIFT scores) are described in Table 4.

**Table 4:** DeepLIFT contribution score orderings for 10 most *predictive* ICD9 codes. DeepLIFT scores were computed using separate test samples and models from ten k-fold cross validation experiments; scores were summed across experiments. DeepLIFT scores were produced for each pair of feature, and output (race and ethnicity) class; we list ten ICD9 codes with the highest ranges of scores—which correspond to discriminative ability. The feature-to-class contribution scores were used to construct orderings of race and ethnicity classes, for each feature. Scores were summed across all samples. Positive scores indicate favorable contribution to a class, zero scores indicate no contribution, and negative scores indicate discrimination against a class.

Rank	ICD9	Description	Ordering of race and ethnicity classes (DeepLIFT scores)
1	401.9	Hypertension NOS	H (961793) > B (723387) > W (330416) > O (102487)
2	789.00	Abdominal pain, unspecified site	H (533874) > B (374665) > W (343653) > O (-45645)
3	V72.6	Laboratory examination	W (385026) > B (-1114) > O (-23509) > H (-86539)
4	V70.0	Routine general medical examination at a health care facility	H (139118) > B (-34600) > W (-35159) > O (-259566)
5	V65.44	Human immunodeficiency virus [HIV] counseling	H (-162191) > B (-248484) > W (-355563) > O (-474608)
6	V76.12	Other screening mammogram	H (535820) > B (425450) > W (414514) > O (253839)
7	V72.9	Unspecified examination	W (313506) > H (212228) > B (193901) > O (68319)
8	V20.2	Routine infant or child health check	H (28390) > B (-68712) > W (-136391) > O (-211301)
9	724.2	Lumbago	H (252024) > B (169995) > W (97782) > O (15679)
10	V72.3	Special investigations and examinations—Gynecological examination	H (-515409) > B (-643566) > W (-665030) > O (-741763)

**Fig 3:**
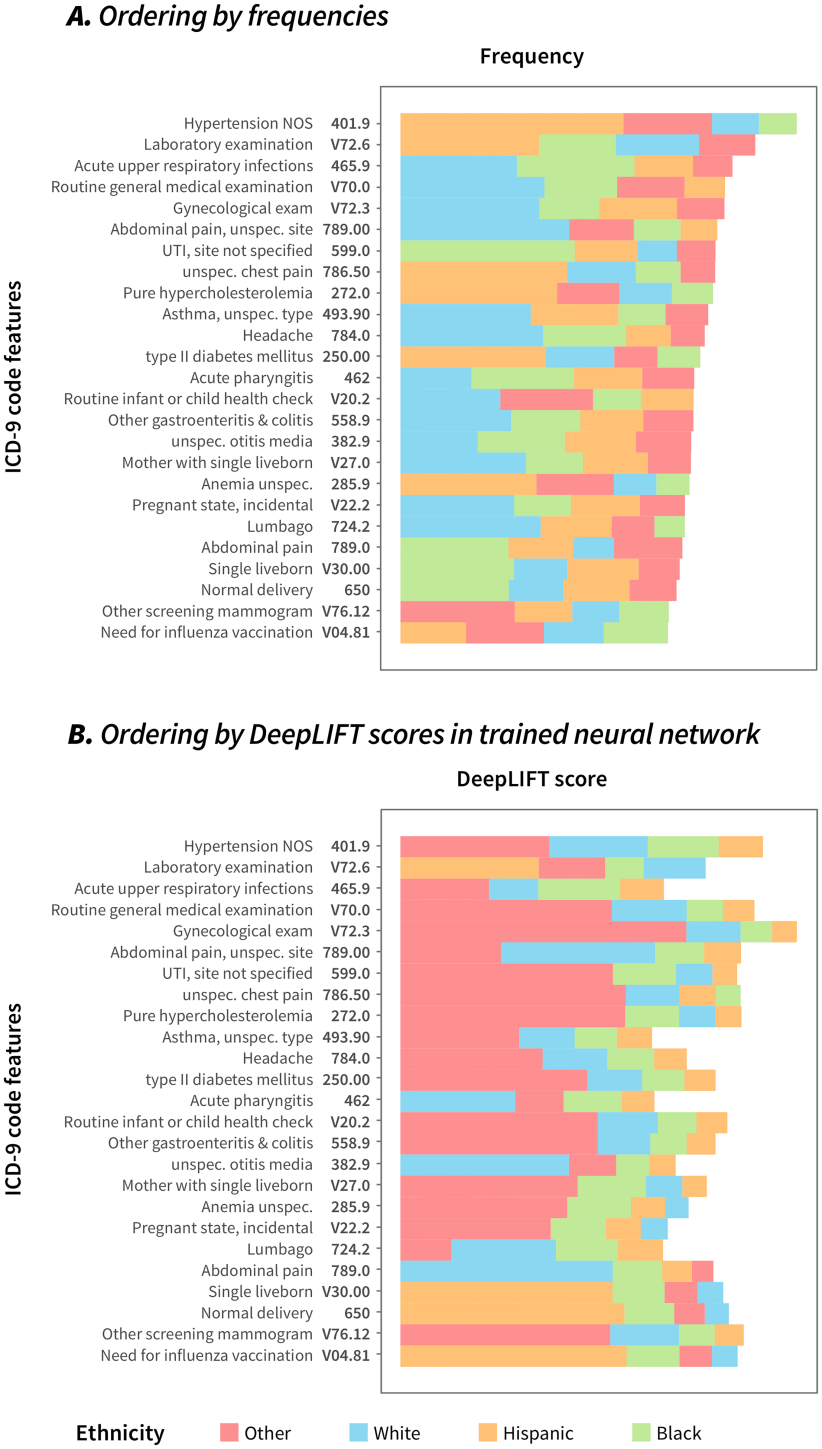
Visualizing class orderings for the 25 most *common* features. We constructed natural orderings of features for the 25 most common ICD9 code features, using (A) frequency information and (B) DeepLIFT scores. Frequency scores were mean-centered; higher scores indicate larger contribution by a feature to a class. These orderings rank the contribution of an ICD9 code to a particular class, and are visualized as a stacked bar. The strongest (positive) feature-to-class contributions are represented by the rightmost bar; the length of the bar corresponds to the magnitude of the contribution on a linear scale. Scores were summed across all samples.

We visualized the orderings of the 25 most *common* features using both frequencies and DeepLIFT scores (see Fig 3; the full table of features is shown in S1 Data). Frequency-based orderings were obtained by sorting the four classes by the number of samples within a class exhibiting a particular feature. Race and ethnicity class orderings obtained from frequency scores were distinctly different than those obtained from DeepLIFT scores. This suggests that RIDDLE’s MLP network is able to learn non-linear and non-frequentist relationships between ICD9 codes and race and ethnicity categories.

According to orderings constructed using DeepLIFT scores, sex is an important feature for predicting race and ethnicity in our models: men who seek medical attention are least likely to be Other followed by African American men. Men who seek medical attention are most likely to be White or Hispanic.

In addition, specific medical diagnoses convey grains of racial and ethnic information: hypertension and human immunodeficiency virus (HIV) are more predictive for African American and Hispanic individuals than White individuals. This finding is also reflected in medical literature, where it has been reported that African American and Hispanic populations are at significantly higher risk for heart disease [11–13] and HIV [14–16] than their White peers.

The fact that these features are important for imputing race and ethnicity could reflect (1) a skewed distribution of blue- and white-collar professions across racial and ethnic groups, (2) uneven accessibility and subjective importance of prophylactic health care across racial and ethnic groups, and (3) possible variation in lifestyle, such as dietary habits. Further work would involve investigating epidemiological hypotheses on how these environmental factors may affect differential clinical patterns across race and ethnicity.

Some of the genetic diseases are famously discriminative for races and ethnicities. For example, sickle cell disease occurs more frequently in African Americans and Hispanic populations than in the rest of the US population [17]. In our model, sickle cell anemia most strongly predicts for the African American and Hispanic classes over the White or Other classes. It has been reported Lyme disease predominately occurs in Whites, and largely unreported for Hispanics or African Americans [18]. This finding is also reflected in our model, where Lyme disease serves as a strong predictor of the White race. Additional strongly White-predictive diseases and medical procedures include atrial fibrillation, hypothyroidism, prostate neoplasm, dressing and sutures, lump in breast, coronary atherosclerosis. These are primarily diseases of older age, suggesting that lifespan varies across race and ethnicity due to socioeconomic and lifestyle reasons, as reported in literature [19, 20].

These orderings provide a high-level description of community structure, and may reflect socioeconomic, cultural, habitual, and genetic variation linked to race and ethnicity across the population of two large cities, New York City and Chicago.

## Discussion

In our experiments, RIDDLE yielded favorable classification performance with class-specific AUC values of above 0.8. Although, RIDDLE uses a fairly simple deep neural network architecture, RIDDLE displayed significantly better classification performance across all tested metrics compared to the popular classification methods logistic regression, random forest and GBDTs. RIDDLE maintained a robust (and significant) classification performance advantage over competitors in experiments simulating missing data. In other experiments, the use of pre-trained bagged embeddings were not helpful to RIDDLE (see Table H in S1 Supplement).

RIDDLE’s superior accuracy and loss results suggest that RIDDLE produces more accurate probability estimates for race and ethnicity classes compared to currently used techniques. Although results could not be obtained for SVMs due to unacceptably high computational costs, RIDDLE significantly outperformed SVMs in runtime efficiency and classification performance on smaller subsets of the full dataset (see Table E in S1 Supplement).

Furthermore, RIDDLE, without the use of a GPU, displayed runtimes comparable to those of traditional classification techniques. With these findings, we argue that deep-learning-driven imputation offers notable utility for race and ethnicity imputation in anonymized EMR datasets. Our current work simulated conditions where ethnicity was missing completely at random. Future work will involve simulating conditions where race and ethnicity are missing at random or missing not at random, and formalizing a multiple imputation framework involving deep-learning estimators.

However, these results also highlight a growing privacy concern. It has been shown that the application of machine learning poses non-trivial privacy risks, as sensitive information can be recovered from non-sensitive features [21]. Our results underscore the need for further anonymization in clinical datasets where race and ethnicity are private information; simple exclusion is not sufficient.

In addition to assessing the predictive and computational performance of our imputation framework, we made efforts to analyze how specific features contribute to race and ethnicity imputations in our neural network model. Each individual feature may represent only a weak trend, but together numerous indicators can synergize to provide a compelling evidence of how a person’s lifestyle, her social circles, and even genetic background can vary by race and ethnicity.

The aforementioned highlights of race- and ethnicity-influenced patterns of health diversity and disparity (see the Results) can be extended to thousands of codes (please see S1 Data for the complete table of features and corresponding annotations). To the best of our knowledge, this systematic comparison across all classes of maladies with respect to race and ethnicity is done for the first time in our study.

## Methods

### Ethics statement

Our study used de-identified, independently collected patient data, and was determined by the Internal Review Board (IRB) of the University of Chicago to be exempt from further IRB review, under the Federal Regulations category 45 CFR 46.101(b).

### Data

We used an anonymized EMR datasets jointly comprising 1,650,000 individual medical histories from the New York City (Columbia University) and Chicago metropolitan populations (University of Chicago). Medical histories are encoded as variable length lists of ICD9 codes (approximately 15,000 unique codes) coupled with onset ages in years. Each individual belongs to one of four mutually exclusive classes of race (Other, White, Black) or ethnicity (Hispanic). Features included quinary gender (male, female, trans, other, unknown), and reported age in years. Age was quantized into discrete categories by integer values.

Onset age information of each ICD9 code was removed and continuous age information was coerced into discrete integer categories. Features were vectorized in a binary encoding scheme, where each individual is represented by a binary vector of zeros (feature absent) and ones (feature present). Each element in the binary encoded vector corresponds to an input node in the trained neural network (see Fig 1).

*k*-fold cross-validation (*k* = 10) and random shuffling were used to produce ten complementary subsets of training and testing data, corresponding to ten classification experiments; this allowed for test coverage of the entire dataset. From the training set, approximately 10% of samples were used as holdout validation data for parameter tuning and performance monitoring. Testing data was held out separately and was only used during the evaluation process.

### A deep learning approach

We used Keras [22] with a TensorFlow backend [23] to train a deep multilayer perceptron (MLP). Neural network architectures and hyperparameters were selected using randomized grid search on 10,000 samples from the validation data. It has been reported that randomized grid search requires far less computational effort than exhaustive grid search with only slightly worse performance [24]. The final neural network hyperparameters are detailed in Table A in S1 Supplement.

The structural architecture of the neural network was fixed across different k-fold partitions prior to training. The neural network was composed of an input layer of 15,122 nodes, two hidden layers of 512 nodes each, and a softmax output layer of four nodes (see Fig 1).

Dropout regularization was applied to each hidden layer with a dropout rate ranging from 0.2–0.8. Dropout regularizes the neural network by randomly dropping neurons and their connections during training; this limits complex co-adaptations between neurons which may not generalize well outside of the training data [25].

For its nodes, our neural network architecture utilizes either Parametric Rectifier Linear Units (PReLUs) [26] or Rectified Linear Units (ReLUs); the choice of which activation to use was determined during hyperparameter tuning.

PReLUs are variants of rectifier functions:

$$\begin{array}{c} f\left(x\right)=\{\begin{array}{cc} x, & x>0;\\ \alpha x, & x\leq 0, \end{array}\;\;\text{where}\;\alpha \;\text{is}\;\text{a}\;\text{learned}\;\text{parameter.} \end{array}$$

where *x* is the input, and *f*(*x*) is the output of the PReLU node. ReLUs are simply PReLUs with the coeficient parameter fixed at *α* = 0.

The MLP was trained iteratively using the *Adam* optimizer [27]. The learning rate, which controls the magnitude of updates during gradient descent, was tuned via randomized grid search. Training was performed in a batch-wise fashion; data vectorization (via binary encoding) was also done batch-wise in coordination with training. The large number of samples (1.65M) and attention to scalability necessitated “on the fly” vectorization. The number of training epochs (passes over the data) was determined by early stopping and model caching [24], where the model from the epoch with minimal validation loss was selected. In order to encourage exploration beyond local minima, a number of epochs with poorer validation loss was permitted in accordance to a fixed patience parameter.

Categorical cross-entropy was chosen as the loss function; categorical cross-entropy penalizes the assignment of lower probability on the correct class and the assignment of non-zero probability to incorrect classes.

### Other machine learning approaches

We evaluated several other machine learning approaches: logistic regression, random forest classifier, gradient-boosted decision trees (GBDTs), and support vector machines (SVMs) with various kernels (linear, polynomial, radial basis function). Traditionally, logistic regression has been used for categorical imputation tasks [8]. We used fast Cython (C compiled from Python) or array implementations of these methods (with the exception of GBDTs) offered in the popular ‘scikit-learn’ library. For the GBDT methods, we used a Python wrapper of the popular XGBoost C library [28].

To handle the multiclass ethnicity imputation problem, we used a one-vs-one implementation of SVMs and a one-vs-all implementation of GBDTs. The implementations of logistic regression and random forest are inherently multiclass. Model hyperparameters were tuned in the same fashion (randomized grid search) as for the deep neural networks. The final hyperparameters are detailed in Tables B-D in S1 Supplement.

### Missing data simulation

In order to replicate real-world scenarios where additional information (other than race and ethnicity) may be absent, we conducted simulation experiments where we randomly removed some proportion of feature data (10%, 20%, or 30%). The number of input features was kept the same as feature observations at the sample level were removed; entire features were not removed.

For example, if 500 patient samples exhibited ICD9 code 401.9 (hypertension NOS) in the training data, we removed, with some fixed probability, the observation of ICD9 code 401.9 for *each* of the 500 individuals. The entire ICD9 code 401.9 feature was not removed—only sample observations of this feature.

We conducted training and testing pipelines with these new “deficient” datasets in the same fashion as before, using ten train/test partitions of the data given by k-fold cross-validation.

The code used to conduct all experiments is available on GitHub (see S1 Code).

### Evaluation

We computed standard accuracy, cross-entropy loss, precision, and recall scores for testing data across all ten experiments. We also computed class-specific ROC AUC scores as well as micro-average and macro-average ROC AUC metrics. Class-specific ROC AUC scores refer to the ROC AUC scores computed by binarizing the classification problem to a specific class. The micro-average ROC AUC score was computed by reducing all multiclass classification problems to binary prediction problems (true class vs. other classes). The macro-average ROC AUC score was calculated by averaging all class-specific ROC scores, weighted by the number of cases in each class.

In addition to evaluating classification performance, we also monitored runtime performance across methods. Models were trained on a standard computing configuration on the Midway compute cluster at the University of Chicago: 16 Intel Sandybridge cores at 2.6 GHz, and 32GB RAM.

Significant differences in performance scores were detected using paired t-tests with Bonferroni adjustment.

### Neural network interpretation

We computed DeepLIFT scores to interpret how certain features contribute to probability estimates for each class [10]. The DeepLIFT algorithm takes a trained neural network and produces feature-to-class contribution scores for each passed sample.

DeepLIFT scores describe how differences in values for some input neuron (compared to a reference value) result in differences in output neuron values (compared to a reference value). The DeepLIFT interpretation method relies on a central summation-to-delta property:

$$\begin{array}{c} \Delta t=\sum_{i=1}^{N}C_{\Delta x_{i},\Delta t} \end{array}$$ (3)

where Δ*t* is the difference-from-reference value for an output neuron. *C*_Δ*x*_*i*_, Δ*t*_ is the difference-from-reference value for the output neuron which can be attributed to differences-from-reference value for a neuron *x*_*i*_ which is necessary to compute the output neuron; this also serves as the DeepLIFT score. Although DeepLIFT does not use gradient information, DeepLIFT scores are computed using a backpropagation-like algorithm which uses a chaining principle analogous to the chain rule. Unlike gradient-based approaches, DeepLIFT scores can be meaningful and non-zero even when the gradient is zero [10].

To compute DeepLIFT scores for the RIDDLE neural networks, we assumed reference values of zeros for all input neurons because our training features were binary and sparse; furthermore, a value of zero for an input feature naturally indicates the absence of a disease state. Alternatively, population statistics for disease incidences could have been used as reference values. Reference values for the hidden layers were obtained by performing a forward pass using input values of zero (the input reference values).

We computed DeepLIFT scores using separate test samples and models from each of our k-fold cross validation experiments to achieve full coverage of the dataset. Scores were summed across experiments for aggregation purposes. To describe high-level relationships between features and classes, we summed scores across all samples to produce an aggregate score. The aggregate DeepLIFT scores for the ten most predictive features are summarized in Table 4.

As described prior, we computed orderings of race and ethnicity classes with each feature’s DeepLIFT scores. These orderings describe how certain features (e.g., medical conditions) can predict for or against a particular race and ethnicity class. We visualize the orderings defined by DeepLIFT scores for the twenty-five most common features in Fig 3, and compare them to the orderings produced from sorting classes by the total number of feature observations within the class. We visualized the orderings of the 25 most frequently observed features in the dataset in Fig 3. For the visualizations, frequency counts were mean-centered to facilitate comparison to DeepLIFT scores.

## Supporting information

S1 Supplement Supporting tables are provided in the attached document supplement.pdf. (PDF)

S1 Data The full list of orderings constructed from DeepLIFT scores is available in the file provided. (TSV)

S1 Code Our implementation of RIDDLE is available as an open-source Python library, riddle. The code is hosted on GitHub, and documentation is available at riddle.ai.
(TXT)
