# Supplementary material for: RIDDLE: Race and ethnicity Imputation from Disease history with Deep LEarning
Source: PLoS Comput Biol. 2018 Apr 26;14(4):e1006106. doi: 10.1371/journal.pcbi.1006106 (PMC5940243; doi:10.1371/journal.pcbi.1006106)
Supplement: S1 Supplement — (PDF) [file pcbi.1006106.s001.pdf]

[*Supplement*] RIDDLE: Race and ethnicity Imputation from Disease history with Deep LEarning

Ji-Sung Kim[1], Xin Gao[2], Andrey Rzhetsky[3*]

1 Department of Computer Science, Princeton University, Princeton, New Jersey, United States of America
2 King Abdullah University of Science and Technology (KAUST), Computational Bioscience Research Center (CBRC), Computer, Electrical and Mathematical Sciences and Engineering (CEMSE) Division, Thuwal, Saudi Arabia.
3 Institute for Genomics and Systems Biology, Computation Institute, Departments of Medicine and Human Genetics, University of Chicago, Chicago, Illinois, United States of America

* andrey.rzhetsky@uchicago.edu

Hyperparameters

We provide values of important hyperparameters found during tuning in Supplementary Tables 1-4.

partition	activation	dropout	learning rate
0	PReLU	0.3005	4.2975×10^{-3}
1	PReLU	0.4473	2.9914×10^{-3}
2	PReLU	0.2244	4.0492×10^{-5}
3	ReLU	0.4181	5.6991×10^{-4}
4	ReLU	0.3522	1.3626×10^{-3}
5	PReLU	0.2731	3.4576×10^{-5}
6	PReLU	0.3195	4.6805×10^{-4}
7	ReLU	0.2535	5.4500×10^{-3}
8	ReLU	0.3513	1.6556×10^{-3}
9	PReLU	0.4850	5.6144×10^{-4}

Table A: Final hyperparameters for RIDDLE. Neural network hyperparameters were determined by randomized grid searches over each k-fold partition. The final hyperparameters for models trained on the full dataset are shown here.

partition	C (regularization strength)
0	0.1075
1	0.1065
2	0.0799
3	0.1036
4	0.0689
5	0.1174
6	0.0872
7	0.0817
8	0.0829
9	0.0803

Table B: Final hyperparameters for logistic regression. Hyperparameters for logistic regression were determined by randomized grid searches over each k-fold partition. The final hyperparameter values for models trained on the full dataset are shown here.

partition	max # of features	max depth	trees
0	sqrt(15122)	90	215
1	sqrt(15122)	69	235
2	sqrt(15122)	102	191
3	sqrt(15122)	121	61
4	sqrt(15122)	59	148
5	sqrt(15122)	93	126
6	sqrt(15122)	80	124
7	sqrt(15122)	76	65
8	sqrt(15122)	126	71
9	sqrt(15122)	113	99

Table C: Final hyperparameters for random forest. Hyperparameters for random forest classifiers were determined by randomized grid searches over each k-fold partition. The final hyperparameter values for models trained on the full dataset are shown here.

partition	max depth	trees	learning rate
0	18	67	0.1267
1	9	149	0.1101
2	7	218	0.0825
3	4	166	0.3848
4	5	129	0.1871
5	8	148	0.0695
6	3	227	0.2095
7	5	245	0.0839
8	9	95	0.1856
9	17	100	0.0954

Table D: Final hyperparameters for gradient boosted decision trees. Hyperparameters for gradient boosted decision trees were determined by randomized grid searches over each k-fold partition. The final hyperparameter values for models trained on the full dataset are shown here.

Additional experiments

We show the results from additional experiments involving a smaller subset of the dataset (see Table E) and various feature selection techniques (see Tables F-G).

Method	Accuracy	Loss	Precision	Recall	F1	Macro-average ROC
RIDDLE	**0.652**	**0.900**	**0.641**	**0.652**	**0.634**	**0.813**
logistic regression	0.636	0.949	0.628	0.636	0.600	0.795
random forest	0.625	0.974	0.629	0.625	0.576	0.782
GBDT	0.629	0.961	0.627	0.629	0.586	0.785
SVM, linear kernel	0.631	0.967	0.629	0.631	0.590	0.791
SVM, polynomial kernel	0.626	0.974	0.616	0.626	0.584	0.838
SVM, RBF kernel	0.643	0.932	0.633	0.643	0.613	0.851

Table E: Evaluation of RIDDLE and baseline methods on 10% of the full dataset. All values are averaged over ten k-fold cross-validation experiments involving a 165k sample subset of the full dataset. In addition, the precision, recall and ROC scores were averaged across classes, weighted by the number of samples in each class.

Method	Accuracy	Loss	Precision	Recall	F1	Macro-average ROC
RIDDLE	**0.648**	**0.902**	**0.655**	**0.648**	**0.618**	**0.814**
logistic regression	0.636	0.949	0.629	0.636	0.598	0.794
random forest	0.634	0.957	0.638	0.634	0.589	0.792
GBDT	0.633	0.953	0.632	0.633	0.590	0.788
SVM, linear kernel	N/A	N/A	N/A	N/A	N/A	N/A
SVM, polynomial kernel	N/A	N/A	N/A	N/A	N/A	N/A
SVM, RBF kernel	N/A	N/A	N/A	N/A	N/A	N/A

Table F: Evaluation of baseline methods trained on the 1000 most frequent features. All values are averaged over ten k-fold cross-validation experiments where only the 1000 most frequent features were used for modeling. In addition, the precision, recall and ROC scores were averaged across classes, weighted by the number of samples in each class.

Method	Accuracy	Loss	Precision	Recall	F1	Macro-average ROC
RIDDLE	**0.654**	**0.896**	**0.652**	**0.654**	**0.629**	**0.812**
logistic regression	0.636	0.954	0.631	0.636	0.598	0.790
random forest	0.636	0.955	0.639	0.636	0.593	0.790
GBDT	0.634	0.953	0.634	0.634	0.592	0.787
SVM, linear kernel	N/A	N/A	N/A	N/A	N/A	N/A
SVM, polynomial kernel	N/A	N/A	N/A	N/A	N/A	N/A
SVM, RBF kernel	N/A	N/A	N/A	N/A	N/A	N/A

Table G: Evaluation of baseline methods trained on the 1000 features with highest chi-squared statistics. All values are averaged over ten k-fold cross-validation experiments where only the 1000 features with highest chi-squared statistics were used for modeling. In addition, the precision, recall and ROC scores were averaged across classes, weighted by the number of samples in each class.

We show the results from using RIDDLE with and without embeddings (Supplementary Table H).

Method	Accuracy	Loss	Precision	Recall	F1	Macro-average ROC
RIDDLE	**0.652**	**0.900**	**0.641**	**0.652**	**0.634**	**0.813**
RIDDLE + embeddings	0.578	1.047	0.584	0.578	0.498	0.810

Table H: Evaluation of RIDDLE without (default) and with pre-trained embeddings on 10% of the full dataset. We evaluated RIDDLE without (default) and with pre-trained embeddings on a 165K sample subset of the full dataset. To use the embeddings with RIDDLE, we mapped ICD9 code features to 605 20-dimension embeddings of bagged ICD9 codes. These embeddings were pre-trained on a large insurance claims dataset. We started with arranging ICD9 codes in each patient history chronologically. In total, we used a collection of 122 million unique patient histories represented in the IBM Watson MarketScan database. These ICD9 codes then were mapped to a smaller set of 605 unique groups (such as asthma or schizophrenia, with each disease group containing multiple ICD9 codes). We then removed repeats of codes in patient histories: for example, no consecutive two asthma codes were allowed. The resulting patient-specific disease sequences were treated as documents, where each patient corresponds to a document, and each disease to a word. We used *gensim* Python module to create the embeddings (context size was 10, minimum count set to 5, and α was changed from 0.001 to 0.0001 in 2×5 iterations) (1). These embeddings were flattened and concatenated to other features not included in the pre-trained embeddings (e.g., age, gender, rare ICD9 codes); the resultant vector was fed as input to the neural network. All table values are averaged over ten k-fold cross-validation experiments. In addition, the precision, recall and ROC scores were averaged across classes, weighted by the number of samples in each class.

References

1. Řehůřek R, Sojka P. Software Framework for Topic Modelling with Large Corpora. In: Proceedings of the LREC 2010 Workshop on New Challenges for NLP Frameworks. Valletta, Malta: ELRA; 2010. p. 45–50. http://is.muni.cz/publication/884893/en.
